# Anti-Counterfeit Technologies: A Pharmaceutical Industry Perspective

**DOI:** 10.3797/scipharm.1202-03

**Published:** 2012-10-09

**Authors:** Dipika Bansal, Swathi Malla, Kapil Gudala, Pramil Tiwari

**Affiliations:** Department of Pharmacy Practice, National Institute of Pharmaceutical Education and Research (NIPER), Sector-67, S.A.S Nagar, Punjab-160062, India.

**Keywords:** Authentication, Anti-counterfeit technology, RFID, Pedigree, Serialization

## Abstract

Growth of international free trade and inadequate drug regulation have led to the expansion of trade in counterfeit drugs worldwide. Technological protection is seen to be the best way to avoid this problem. Different technologies came into existence like overt, covert, and track and trace technologies. This review emphasises ideal technological characteristics, existing anti-counterfeit technologies, and their adoption in different countries. Developed countries like the USA have implemented RFID while the European trend is towards 2D barcodes. The Indian government is getting sensitised about the extent of the problem and has formulated rules mandating barcodes. Even the pharmaceutical companies have been employing these technologies in order to detain illegitimate drugs in their supply chain.

## Introduction

A medical product is counterfeit when there is a false representation in relation to its identity or source. This applies to the product, its container, or other packaging or labeling information. Counterfeiting can apply to both branded and generic products and counterfeit products may include products with the correct or wrong ingredients, without active ingredients, with incorrect amounts of active ingredients, or with fake packaging [1]. Less developed countries were found to be major contributors to the counterfeit medicines [2]. Counterfeit medicines pose significant health risks to patients which results in unnecessary morbidity and mortality. At 10% of global trade, counterfeiting affects many countries, causing serious downstream expenses and resource shortages [3]. Many classes of drugs have been counterfeited and several factors are associated with it like poor regulation, a higher number of intermediaries, lack of awareness, and many more [4]. Drug counterfeiting is likely to increase in the future as health care costs continue to rise around the globe. Globalization trends and deregulation continue to open new markets by which demand for pharmaceuticals is likely to increase, and with it the supply of counterfeit drugs [5]. The anti-counterfeiting measures include the methods, techniques, devices, and nanomaterials which aid in the protection of the supply chain of industrial and consumer goods [6].

The security of the pharmaceutical supply chain can be strengthened by innovative packaging technologies and better business practices [7]. Currently, Indian pharmaceutical companies produce about 20–22% of the world’s generic drugs (in value terms) and therefore counterfeiting is a subject that has great relevance for the Indian industry [8].

## Need for anti-counterfeit technologies

Counterfeit drugs can lead to drug recalls and liability suits. In addition, brand loyalty is compromised as consumers perceive additional risks when using a company’s products. An effective anti-counterfeit strategy avoids this and ensures patient safety [9]. Principle ways of combating counterfeiting include: legal actions on illicit traders, countermeasures using technologies, consumer education and information, private investigations, and cooperation with enforcement agencies. The implementation of anti-counterfeit technologies is the prominent preventive measure [9]. In addition to providing authentication, they make the production of a convincing copy of a drug more difficult and costly [7]. The government authorities, by employing these technologies, may ensure that drugs in the supply chain are legitimate. For example, the US Prescription Drug Marketing Act of 1987 (PDMA), amended by the Prescription Drug Amendments of 1992 (PDA), requires wholesalers to provide a pedigree prior to each wholesale distribution of prescription drugs [10].

## Characteristics of Ideal Anti-counterfeit Technology

An ideal anti-counterfeit technology should possess a high level of security (non-clonable), higher product application and authentication speed, proven standards, be difficult to remove and reapply, easy to check, have automatic authentication, be useable by consumers, and must be legally compliant by the industries [11]. However, the FDA recommends the use of multiple, periodically changing, authentication measures on a product-specific basis [12].

## Overview of Current Anti-counterfeit Technologies

Multiple anti-counterfeit technologies with distinct advantages and drawbacks exist today [13, 14]. Primarily, these can be used in three different ways:

### Tamper-evident/tamper-resistant packing

Packaging having an indicator or barrier to entry which, if breached or missing, should provide visible or audible evidence to consumers that tampering has occurred [15]. Eg. film wrappers, shrinkable seals and bands, breakable caps, tape seals, blister packs, etc.

### Product authentication

Authentication features can be embedded either on the dose or on packaging of the medicines. These are overt / covert / forensic features shown in Table 1[16].

#### Holograms for Anti-counterfeiting

Holograms can combine three layered security features and become a most powerful weapon against counterfeiting. In such solutions, holograms can provide overt first line authentication while covert features such as scrambled images, microtext, UV-sensitive or other specialised inks provide second line authentication for trained examiners and appropriate decoding equipment. Serialization of holograms is another trend that combines authentication with traceability [17]. Some of these developed technologies are binary encrypted holograms, light diffraction hologram elements in a product label, or a combination of a hologram, 2D datamatrix, and thermal monitoring [18, 19].

### Track and trace technology

This is the process of assigning a unique identity to each stock unit during manufacture which then remains with it through the supply chain until its consumption, and is called the track and trace system. Information is attached in the form of a unique pack coding, enabling access to the same information on a secure database [8].

#### Pedigree

This is a type of track and trace system of the drugs in a location. In the US, the final pedigree regulations were drafted in 1999 for PDMA [20]. A drug pedigree is a paper document or electronic file that records the details of distribution of a prescription drug from its manufacture through wholesale transactions, until it is received by the dispenser, which is usually a pharmacy or physician. The person who receives a pedigree along with the drug shipment must verify that each recorded distribution took place and that the drug-specific information (such as lot number and manufacture date) is correct. This system of pedigree passage and authentication is intended to ensure that prescription drugs cannot easily be diverted or replaced with counterfeit products [21]. The electronic system has largely replaced the paper system because of disadvantages like incompatibility with the bulk of pharmaceuticals, record keeping failures and fraud, and higher probability of counterfeit with paper pedigree [22].

Several other advantages are offered by the track and trace system like a reduction in the number of medication errors, automated pharmacy billing, effective inventory control, facilitation of product recalls, and identification of theft and product diversion [23].

#### Mass Serialization

Serialization includes the processes of generating, encoding, and verifying the unique identity of individual physical items [24]. Without mass serialization, the authenticity and validity of the pedigree relates only to the lot number consisting of thousands of bottles. However, a specific bottle of a particular drug cannot be authenticated [25]. When combined with track and trace technology, serialization facilitates the tracking of a product through the supply chain and allows for targeted identification of products for recall [24]. Global Standards one (GS1) is a not-for-profit organization that develops global standards for the identification of goods and services. GS1 standards are used for the identification of pharmaceutical products in 60 countries around the world [26]. The GS1 identifiers related to the pharmacy supply chain are:

#### Global Trade Item Number (GTIN)

A 14/13/12/8 digitally unique identification number is assigned by the manufacturer in accordance with GS1 allocation rules for trade items or products and services. It is constructed from a company prefix assigned by GS1, an item reference number designated by the company, and a check digit [24]. Prior to market requirements for item-level serialization and verification, Astrazeneca made it as an internal initiative [27].

#### Serialized Global Trade Item Number (sGTIN)

A unique number that identifies a particular trade item, created by appending a serial number to the GTIN of the product. In their draft guidance for serialized identifier prescription drugs, the FDA proposed the use of the NDC (which forms part of the GTIN in the US realm) combined with an eight-digit serial number [24]. In March 2010, the FDA issued guidance addressing the package level SNI (Serialized Numerical Identifier) to be “applied to a prescription drug at the manufacture and repackaging of the product to facilitate its track and trace” [28].

The main challenges of implementing serialization are the complexity of data that is to be tracked, and the need for potentially huge, multi-access databases [29].

#### Data Carriers

Data carriers are graphical systems used to convey the product identifiers and associated information in computer and/or human readable format. A mark, tag, or label applied at the source represents them. Computer readable formats include linear and two dimensional (2D) bar codes and radio frequency identifier (RFID) tags [24]. These are the most frequently employed nowadays (Table 2) [30].

#### Multi-level approach

Anti-counterfeiting technological approaches are interdependent for their effectiveness, and integrating them yields a more robust system. In this respect, a combination of overt and covert measures may provide optimal security because they help prevent counterfeiting and reassure end-users [29]. For example, using drug product serialization in combination with electronic pedigree greatly increased the level of security by the ability to verify both the product and the transaction integrity [31]. For example, the serialization of holographic labels [29]. Some organizations such as Authentix and Nosco have made initiatives to combine the respective limitations and the potential of both Data Matrix and RFID, such that cases and pallets can be tracked with RFID tags, while medicines can be tracked with Data Matrix [32]. However, a multi-level approach may also result in additional costs as the technologies become more sophisticated and should be implemented based on the risk analysis of the drug to be counterfeited.

## Markets of Anti-Counterfeiting Technologies

BCC Research [33] showed that the global sales of anti-counterfeiting packaging technologies were worth US $64 billion in 2010, and the value is projected to be $74.2 billion in 2015. Track and trace technologies and authentication technologies made up for sales of US$31.7 and $32.4 billion respectively in 2010. Expected values of these by 2015 are US$36.5 and $37.7 billion respectively [33].

## Recent Technologies

Use of isotope excipients: A recent study showed the ability of labeled excipients in different ratios to provide in-product, batch-specific identification using existing technology in the pharmaceutical industry [34]. For adoption in developing countries like India, a system named epothecary was proposed which is a mobile-based authentication system. This is a cost-effective approach with minimum requirement of network infrastructure [35]. A research study proposed the application of semiconductor nanostructures called quantum dots as a promising strategy against counterfeiting [36].

## Anti-counterfeit measures in the pharmaceutical industry

Several anti-counterfeit technologies are being utilized by pharmaceutical firms to prevent the counterfeits. Some of the products with the corresponding anti-counterfeit measures are mentioned in Table 4. Along with the anti-counterfeit technologies, the analytical techniques are also used by pharmaceutical industries for authentication which are explained in a later section.

## Overview of Analytical Techniques

Authentication using the various anti-counterfeit technologies can verify the supply chain, while analytical methods like chromatography, optical spectroscopy, and isotopic characterisation confirm the composition [41]. Several analytical techniques were being employed by different pharmaceutical industries for the detection of counterfeits. They include simple techniques like colorimetry and thin-layer chromatography, to advanced methods like NMR, mass, and raman spectroscopies [42]. Handheld devices are currently gaining more importance and numerous instruments are being developed. Mainly portable devices like the handheld raman and IR spectrometers are being utilized owing to their non-destructive, rapid, and reliable properties. An increased demand for these is seen not only by the pharma companies, but also by health authorities, regulators, and law enforcement agencies. Some of the reasons owing to such demand are that these are rapid, indestructible, portable, and easy to use by non-experts. There are also other handheld devices like the X-ray and mid IR spectrometers [43]. Some of these devices developed by different firms are mentioned in the following table.

## Global Implementation of Anti-counterfeit Technologies

According to the Healthcare Distribution Management Association (HDMA) as of July, 2008, 29 states in the US had implemented pedigree requirements and six other states had pending legislation. Federal law requires all dealers except manufacturers and ADRs to pass pedigree requirements, but state requirements show several variations. A limited number of transactions are allowed without the pedigree requirement, as in Nevada: pedigrees must be passed when a drug leaves a normal distribution chain (as is the case in Arizona, Maryland, and North Dakota), and in Florida where all wholesale distributors including authorized distributor of records (ADR) must comply with the law. The new deadline for drug manufacturers’ initial phase of compliance with California’s serialization and pedigree requirements is January 1, 2015 and the remaining 50 percent (or less) of their drug products is no later than January 1, 2016. Wholesale distributors and repackagers must bring their systems to be compliant by July 1, 2016, while pharmacies and pharmacy warehouses must by July 1, 2017 [21].

The Mobile Authentication Service is a National Agency for the Food, Drug Administration, and Control (NAFDAC) program in Nigeria supported by BIOFEM pharmaceuticals where Sproxil technology enables any consumer to check the authenticity of medication with a simple text message [44]. GSK with NAFDAC collaboration uses this service for the authentication of the antibiotic ampiclox (tradename) [45].

The “Meditag” holographic authentication sticker was introduced in 2005 by the Malaysian Ministry of Health to confirm the authenticity of medicines registered [46].

According to a French Official Journal dated March 16, 2007, the French Health Products Safety Agency notified distributors mandating the following in their healthcare system by the 31st of December in 2010 [47]: a 13-character (Club Inter Pharmaceutique) CIP 13 code with the batch number and expiration date and Simplex linear barcode to be replaced by the 2D Data Matrix marking.

The Automated Identification of Vaccine Projects (AIVP) Advisory Task Group of Canada as documented in the Canadian Consensus Statement released in late 2009, stated the following recommendations for all vaccines in Canada [48]: 2D bar codes on the primary package and 2D or linear barcodes on the secondary package, both of which include the GTIN and the lot number.

### Indian Scenario

According to the public notice issued by the Directorate General of Foreign Trade dated January 10^th^, 2011, exported pharmaceutical products should have track and trace capability using barcode technology as per GS1 global standards [49]. The stated requirements are: 2D barcode at the primary level, 1D or 2D on the secondary level, and 1D at the tertiary level packaging encoding the GTIN code, batch number, expiration date, and serial number of respective packaging. However, this system does not ensure the absence of counterfeits as effectively as serialization. Barcodes increase the risk of being caught if counterfeits are present, whereas serialization uniquely identifies every entity and ensures the absence of counterfeits. Serialization using barcodes as data carriers is a more secure strategy and is even more economical compared to the RFID system.

## Drawbacks of anti-counterfeit technologies

Counterfeit-resistant technologies must be rotated regularly as they can themselves be duplicated, often within 12–18 months. Neither pharmacists nor patients can be expected to be aware of the wide range of overt features if they are rotated on a regular basis. Overt and covert packaging technologies are rendered useless if a drug is repackaged [50].

## Educational Programs

Every stakeholder is crucial in combating counterfeiting, as counterfeit drug prevention is a collective job. Healthcare professionals as well as patients should be vigilant about the medicines procured and their source. They should evaluate the response, educate others regarding inspection of the authenticity of the drug acquired, and report in the case of suspicion. Business personnel must be educated about the implementation of good anti-counterfeit technology and make consumers aware of such strategies used. Regulatory authorities must conduct checking plans and devise necessary measures to ensure the absence of counterfeits, increasing the penalty of the pharmaceutical counterfeiting based on the risk imposed on public health [7].

## Conclusion

Drug counterfeiting is an important problem addressed by several countries. This requires multiple measures to protect the supply chain. The implementation of anti-counterfeit technologies is an important strategy taken up by several drug manufacturers and regulatory authorities. The track and trace system and serialization are given importance and are widely used among all anti-counterfeit technologies in different countries. Recent notification from the Indian government mandated the use of barcodes on all drug products manufactured and imported.

## Figures and Tables

**Fig. 1:**
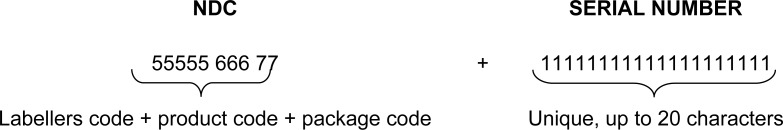
Example of a serialized national drug code

**Tab. 1.** Comparison of Authentication Characteristics

**Features**	**Overt features**	**Covert features**	**Forensic features**
Examples	Holograms, colour-shift Inks	Embedded images, digital watermarks, invisible printing	Chemical and biological tags, microtaggants
Advantages	User verifiable, more secure, decorative appeal, low cost	Easily added or modified, need regulatory approval, applied in-house or via component suppliers, low cost	High-tech and secure against copying, provide positive authentication, may be disclosed for overt purposes
Disadvantages	Require user education, easily mimicked, rely on covert features for authentication, may be re-used or refilled, provide false assurance	Need strict secrecy, risk of compromise, more secure options add supply complexity and cost	Licensed technologies, significant cost, difficult to implement and control across many markets, unlikely to be available to authorities or public

**Tab. 2.** Radio Frequency identifier (RFID) Vs 2 Dimensional (2D) Barcode

**Features**	**2D BARCODE**	**RFID**
Direct line of sight requirement	Yes	No
Difficult to duplicate or alter	No	Yes
Readability robustness (interference with liquids/metals)	No	Yes
Cost of tags	Low	High
Tag data storage	Low	High
Bulk tag reading	No	Yes
Initial technology set up cost	Low	High
Eco-system and/or standards maturity	High	Medium
Tag feature’s extendibility (ex. Tag with sensors)	Low	High

**Tab. 3.** Patented Anti-counterfeit Technologies [37, 38]

**Company Name**	**Technology**	**Description**	**(Country) Patent Number**
Microsoft Corporation	Labels using randomly-occurring features	Pattern unique to each label	(US) 7,878,398
Axsun Technologies	Taggants read using Raman spectroscopy	Can be made unreadable by chemical modification	(US) 7,875,457
CSEM SA (Centre Suisse d’Electronique et de Microtechnique) (Swiss firm)	Zero-order diffractive pigments (ZOPs)	Produce very pronounced colours that can’t be copied	(US) 7,864,424
AlpVision (Swiss firm)	Cryptoglyph invisible marking technology	Invisible signature on package	(India) 243454 (Indonesia) P0025514B
PANalytical BV (Dutch firm)	Use of angle-dispersive X-ray diffraction	Compared to reference signatures in data library	(US) 7,756,248

**Tab. 4.** Anti-counterfeit technologies used in brand pharmaceutical products [39, 40]

**Pharmaceutical Company**	**Product**	**Active Ingredient**	**Authentication Mark**
Pfizer Inc.	Viagra^†^	Sildenafil citrate	RFID
Hoffmann-La Roche Ltd	Rocephin^†^	Ceftrioxone sodium	Logo stamps inside vials
GlaxoSmithKline Pharmaceuticals Ltd	Trizivir^†^	Abacavir/lamivudine/zidovudine	RFID
Purdue pharma L.P.	Oxycontin^†^	Oxycodone HCl	RFID

† Brand names.

**Tab. 5.** Examples of portable devices [43]

**Analytical method**	**Device name**	**Firm**
Raman spectroscopy	TruScan RM	Thermo Fisher Scientific
Near IR spectroscopy	MicroPhazir	Thermo Fisher Scientific
Visible/near-IR spectrometer	LabSpec	The Boulder
FTIR spectroscopy	Multipurpose analyzer Lumos	Bruker optics
FTIR spectroscopy	Exoscan 4100 spectrometer	Agilent technologies
